# Adrenocortical and Adipose Responses to High-Altitude-Induced, Long-Term Hypoxia in the Ovine Fetus

**DOI:** 10.1155/2012/681306

**Published:** 2012-05-14

**Authors:** Dean A. Myers, Charles A. Ducsay

**Affiliations:** ^1^Department of Obstetrics and Gynecology, University of Oklahoma Health Sciences Center, Oklahoma City, OK 73104, USA; ^2^Center for Perinatal Biology, Loma Linda University School of Medicine, Loma Linda, CA 92350, USA

## Abstract

By late gestation, the maturing hypothalamo-pituitary-adrenal (HPA) axis aids the fetus in responding to stress. Hypoxia represents a significant threat to the fetus accompanying situations such as preeclampsia, smoking, high altitude, and preterm labor. We developed a model of high-altitude (3,820 m), long-term hypoxia (LTH) in pregnant sheep. We describe the impact of LTH on the fetal HPA axis at the level of the hypothalamic paraventricular nucleus (PVN), anterior pituitary corticotrope, and adrenal cortex. At the PVN and anterior pituitary, the responses to LTH are consistent with hypoxia being a potent activator of the HPA axis and potentially maladaptive, while the adrenocortical response to LTH appears to be primarily adaptive. We discuss mechanisms involved in the delicate balance between these seemingly opposing responses that preserve the normal ontogenic rise in fetal plasma cortisol essential for organ maturation and in this species, birth. Further, we examine the response to, and ramifications of, an acute secondary stressor in the LTH fetus. We provide an integrative model on the potential role of adipose in modulating these responses to LTH. Integration of these adaptive responses to LTH plays a key role in promoting normal fetal growth and development under conditions of a chronic stress.

## 1. Introduction

Hypoxia represents a major threat to the developing fetus and often accompanies situations of preeclampsia, preterm labor, smoking, and high altitude. The impact that hypoxia has on the fetus depends on a variety of factors such as stage of gestation, severity of the hypoxic episode, duration of hypoxia, and the association with other confounders such as acidemia and hypercapnia. Severe hypoxic events can lead to fetal death or exert major deleterious impacts on development and function on fetal organs, including major damage of the CNS. However, fetuses are often and perhaps routinely exposed to less severe hypoxic episodes. While these hypoxic conditions may impact fetal growth, effects on other organ systems are less clear.

 Due to its long gestational length, similarity of endocrine and physiological systems, and relative ease of fetal and maternal instrumentation, the sheep has emerged as a major animal model for studying the impact of hypoxia on the developing fetus. These studies have largely focused on so-called acute hypoxia in which a moderate-to-severe hypoxia (a decrease in fetal PO_2_ of 50% or greater) is induced for times ranging from minutes to several hours or chronic hypoxia in which the fetus is made hypoxic for one to several days. Experimentally, acute fetal hypoxia can be induced by maternal hypoxia [1–3], restricting blood flow to the uterus and placenta on the maternal side [4], or reducing umbilical blood flow by cord compression [5–7]. More prolonged fetal hypoxemia can also be initiated by repeated umbilical cord compression [7–9], as well as placental embolization [10, 11] and restriction of uterine blood flow [12]. These methods induce either a rather pure hypoxic episode or hypoxia accompanied with acidemia or hypoglycemia. Longer duration hypoxic situations (months) have been created in sheep by maternal carunclectomy, reducing the placentation area of the uterus [13]. However, the hypoxia in this model is secondary to nutrient restriction.

Our model of long-term hypoxia (LTH) uses pregnant ewes maintained at high altitude (3,820 m, Barcroft Laboratory White Mountain Research Station, Bishop, CA) from approximately 40 days' gestational age onward resulting in a maintained fetal PO_2_ of ~18 mmHg (normal ~23–25 mmHg) thus resulting in a *moderate* state of continuous hypoxia [14–16]. Under these conditions, fetal hypoxia is achieved without accompanying acidosis, the pregnancies are of normal duration, and the fetuses do not exhibit growth restriction [15, 17]. Considering that hypoxia is a potent fetal stressor even at the moderate level attained in our model of LTH, the ability of the LTH fetus to mount an adaptive response circumventing preterm birth and growth restriction is remarkable. The focus of our studies has been the adaptive responses of the fetal hypothalamo-pituitary adrenocortical (HPA) and adrenomedullary axis. During our studies on these components of the stress response, we have also initiated studies into how LTH impacts development of the fetal perirenal adipose tissue and interactions between this tissue and the HPA axis.

## 2. Effects of LTH on the Fetal HPA Axis

 Since hypoxia is a potent activator of the fetal HPA axis, we initially explored the effect of LTH on the systemic endocrine HPA response by quantifying basal immunoreactive (IR) ACTH and cortisol concentrations in fetal plasma as well as the response of LTH fetal sheep to an acute secondary stressor (severe hypoxia via hypotension or umbilical cord occlusion: UCO) during the later stages of gestation [14, 16, 18]. Surprisingly, despite the continued stress of LTH, fetal plasma cortisol concentrations were not elevated in these fetuses compared to normoxic control fetuses at 136–141 days gestation (term is ~145 days). Thus, the LTH fetus maintains a seemingly normal late gestation maturation of the fetal adrenal cortex and thus preserves the late gestation exponential rise in cortisol that precipitates organ maturation and, in this species, birth. Similar to cortisol, fetal plasma concentrations of IR-ACTH were also similar in the LTH fetus compared to normoxic controls. Thus, by initial observations, these fetuses apparently adapt to LTH such that this chronic hypoxic perturbation is no longer perceived by the fetus as stressful.

Paradoxically, the HPA axis response to an acute secondary stressor such as hypotension or UCO [14, 16] is decidedly different than predicted based on the basal circulating levels of IR-ACTH and cortisol. Indeed, we found in response to these acute stressors that fetal plasma cortisol was elevated in the LTH fetuses compared to normoxic controls while concentrations of fetal plasma IR-ACTH achieved were similar in LTH and normoxic fetuses. These findings implicated a potential intra-adrenal mechanism(s) that allow the adrenal cortex of the LTH fetus to respond to an acute stress with increased cortisol production while maintaining the normal basal ontogenic maturation.

 Neural input has been shown to play a physiological role in regulating cortisol production. For instance, bilateral section of the splanchnic nerve reduces cortisol production in response to endogenous or exogenous ACTH [19, 20] while stimulation of the splanchnic nerves enhances cortisol output [21, 22]. In near term normoxic fetal sheep, it has been reported that sinoaortic denervation [23] or splanchnic nerve section [24] decreased the cortisol response to *acute* stress without affecting stress-induced ACTH release. However, in the LTH fetus, the enhanced cortisol response to a secondary stressor does not appear to be mediated by neural input to the adrenal cortex since adrenal denervated LTH fetuses responded in a manner similar to the intact LTH fetuses [25]. Thus, the increased response of the adrenal cortex to an acute secondary stress in the LTH fetus does not emanate from a neurally mediated mechanism. Based on these initial observations, we further explored the origin(s) of the observed adaptive responses of the HPA axis to LTH at the level of the hypothalamus and anterior pituitary as well as the adrenal cortex.

## 3. Effect of LTH on the Hypothalamus and Anterior Pituitary

Increases in the bioactivity of plasma IR-ACTH have been noted in late gestation fetal sheep, both in terms of ontogeny and in response to an acute stressor [26–29]. In adults, the majority of circulating IR-ACTH consists of fully processed ACTH_1–39_. However in the ovine fetus, a considerable proportion of IR-ACTH is the unprocessed ACTH precursor, proopiomelanocortin (POMC), or the so-called 22 kDa proACTH, which is the partially processed N-terminal region of POMC through the carboxyl terminus of ACTH [30, 31]. In addition, a few other POMC-derived products containing the ACTH peptide, likely produced via incomplete and/or improper processing at the anterior pituitary or lung [32, 33], are also found in the fetal circulation. Both POMC and 22 kDa proACTH have been noted to inhibit ACTH-stimulated cortisol production or exhibit week ACTH bioactivity [34]. These precursors exhibit varying degrees of cross-reactivity in classical one-site ACTH radioimmunoassays or ELISAs thus giving rise to the concept of bioactivity of IR-ACTH. Therefore, during late gestation the processing of POMC to ACTH in the anterior pituitary corticotrope matures and this maturation is largely governed by the hypothalamic paraventricular nucleus. This was confirmed by our observation that stereotaxic lesion of the fetal PVN, the source of the two major corticotrophin releasing factors, CRH and AVP, prevents the late gestation increase in POMC processing to ACTH [35]. Thus, we hypothesized that LTH, acting as a chronic stressor, may have enhanced POMC processing to ACTH in the fetal anterior pituitary.

**Figure 1:**
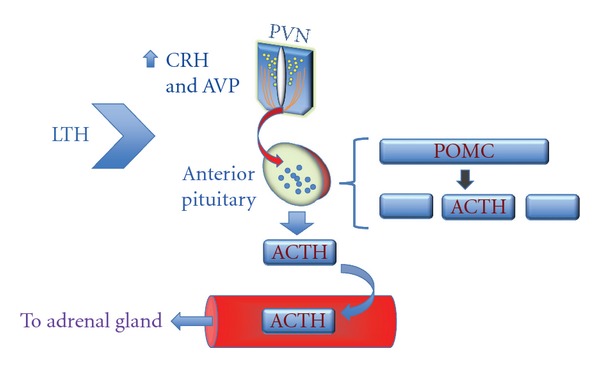
Effects of development under conditions of long-term hypoxia (LTH) on hypothalamic PVN and anterior pituitary corticotrope function in the late gestation ovine fetus. LTH upregulates expression of both CRH and AVP in the parvocellular division of the PVN. At the level of the anterior pituitary corticotrope, this increased hypothalamic drive results in increased processing of POMC to ACTH as well as ACTH release from the gland into the circulation. The changes at the level of the PVN and anterior pituitary result in increased basal circulating ACTH as well as an enhanced ACTH release in response to a subsequent acute stressor.

To evaluate this possibility, we analyzed both basal and stress-induced ACTH_1–39 _and ACTH precursor concentrations in fetal plasma of LTH compared to control fetuses using a two-site IRMA specific for ACTH_1–39_ and an ACTH precursor IRMA [36]. In response to an acute secondary stressor, UCO, fetal plasma concentrations of ACTH_1–39_ were higher in the plasma of LTH fetuses compared to similarly stressed normoxic fetuses. Although ACTH precursors were also elevated, it was to a lesser extent than ACTH and thus the ratio of ACTH_1–39_ to precursors was increased in the LTH compared to normoxic fetuses, indicating that the bioactivity of fetal plasma IR-ACTH achieved in the LTH fetuses was greater than that in normoxic fetuses (see Figure 1, [37]). This is consistent with the enhanced cortisol response observed in LTH fetuses during this secondary stressor. Intriguingly, basal plasma ACTH_1–39_ concentrations were also elevated in the LTH fetal sheep. Despite the elevated plasma levels of ACTH_1–39_, these fetuses maintain normal basal cortisol concentrations compared to normoxic control fetuses.

To support the elevated plasma ACTH_1–39_, we found enhanced processing of POMC to ACTH in the anterior pituitary of the LTH fetus, as well as signs of increased secretion (less stores) accompanied by sustained POMC expression with a trend toward greater POMC mRNA [36]. Considering our observations that lesion of the PVN prevents the enhanced processing of POMC to ACTH during late gestation [35], we hypothesized a hypothalamic origin for the increased processing of POMC in response to LTH. In our original report [36], we noted only a trend for hypothalamic CRH mRNA to be increased in the LTH fetuses using real-time PCR analysis of whole dissected hypothalami. However, recently (Ducsay and Myers, unpublished observations) using in situ hybridization techniques, we found a significant upregulation of both CRH mRNA and AVP mRNA in the hypothalamic parvocellular division of the PVN. Initial morphometric analysis also suggests an expansion of the PVN reflecting an increase in the number of CRH neurons or neurons expressing CRH at detectable levels in response to LTH.

 At the level of the anterior pituitary, we noted increased mRNA for the CRH_1_ receptor with decreased CRH_1_ protein- the latter perhaps suggestive of a downregulation of this receptor in response to increased hypothalamic drive to the anterior pituitary. Of note, anterior pituitary AVP receptor (V1b) expression (mRNA and protein) was upregulated in the LTH fetus perhaps indicative of a selective gain of responsiveness to this ACTH secretagogue [18]. In support of this potential gain of AVP function in response to LTH, we also found that ACTH release in response to AVP was greater in LTH fetal sheep, while the response to CRH was similar between LTH and normoxic controls. Thus, the hypothalamic and anterior pituitary corticotrope response to LTH appears complex and may represent a shift from CRH to AVP in the regulation of the HPA axis.

It appears that LTH advances the normal ontogenic maturation of POMC processing to ACTH at the anterior pituitary via actions at the level of CRH and more likely, AVP neurons, in the parvocellular PVN (Figure 1). Upon casual observation, while this helps to explain the increased cortisol response to a secondary stressor in the LTH fetus, it is in conflict with the noted maintained normal basal plasma cortisol levels in these fetuses. Importantly, the fetal adrenal cortex seems to have developed an adaptive mechanism preventing an early maturation culminating with a premature rise in fetal plasma cortisol resulting in a growth restricted, preterm fetus. Thus, we shifted our focus to the adrenal cortex.

## 4. Effect of LTH on the Ovine Fetal Adrenal Cortex

Based on the elevated basal plasma ACTH_1–39_ noted in the LTH fetuses and the capacity for greater cortisol production in response to a secondary stressor, we initially hypothesized that the LTH adrenal cortex would have responded correspondingly with an increase in expression of key enzymes regulating cortisol biosynthesis as well as supporting genes such as StAR and the ACTH receptor. However, despite the elevated basal plasma ACTH_1–39_, expression of two key enzymes mediating cortisol synthesis (CYP11A1 and CYP17) was approximately 50% lower in the LTH adrenal cortex as was the ACTH receptor [38]. Although we initially did not observe any change in StAR mRNA, we did note an increase in the 30 kDa spent form of the cholesterol transporter protein. An increased transport of cholesterol into the inner mitochondrial membrane, the site of the first limiting step in cortisol biosynthesis via CYP11A1 could help maintain basal plasma levels of cortisol noted in LTH fetuses despite the noted lowered expression of the steroidogenic enzymes.

The findings of decreased, not increased, levels of genes whose expression is exquisitely dependent upon ACTH in the LTH adrenal cortex led to a key fundamental question: namely, what LTH-stimulated mechanism(s) would provide essentially a brake on early activation and excessive production of cortisol, preventing early birth and growth restriction? Initially, we analyzed expression of a key transcription factor, SF-1, via which ACTH coordinates expression of its responsive genes in the adrenal cortex. Levels of SF-1 as well as DAX-1, a transcription factor repressing expression of CYP17 and CYP11A1, were not altered in the LTH adrenal cortex [38]. Since these factors also play a key role in adrenocortical differentiation and growth, this is consistent with no differences in adrenal zonation or size in the LTH fetus, but not with the decreased expression of the downstream target genes. We also examined expression of factors (pMEIS-1 and Pbx-1) that act in concert with SF-1 in the bovine adrenal cortex and thus presumably the highly conserved regulatory regions of the ovine CYP11A1 and CYP17 promoters. Similar to SF-1, these factors were also expressed to a similar degree in LTH compared to normoxic adrenal cortices (Ducsay and Myers [39], unpublished observations). Thus, the effects of LTH on the transcription of the ACTH-receptor, CYP11A1, and CYP17 must likely be either through an unknown repressor or, more likely, via altered activation of these transcription factors. It has been demonstrated that SF-1 activity is regulated by phosphorylation state, dictated by either ACTH activated cAMP/PKA pathways or ERK1/2 pathways, and dependent on protein phosphatase activity [40].

To address the potential for a decreased second messenger response to ACTH, especially in light of the decreased ACTH receptor expression, we examined cAMP production in adrenocortical cells obtained from LTH fetuses. Surprisingly, LTH fetal adrenocortical cells generate a similar cAMP response compared to adrenocortical cells from normoxic fetuses [41]. In these in vitro studies, we also noted an additional perplexing finding: that contrary to the in vivo situation, LTH fetal adrenocortical cells exhibited greater basal cortisol production, while retaining the noted enhanced ACTH stimulated cortisol output compared to cells from normoxic fetuses. A similar enhanced cortisol response to cAMP was also noted, thus emphasizing that the effect was potentially downstream from second messenger generation.

Thus, there do not appear deficiencies in ACTH signaling via the major second messenger pathway utilized by the ACTH receptor. Thus, the noted lower mRNA levels for the ACTH receptor may not be reflected by actual levels of the ACTH receptor protein or that levels of the ACTH receptor may not be limiting. Alternatively, lower ACTH receptor expression may be overcome through more efficient coupling of the ACTH receptor to G*α*s or increased adenylate cyclase activity. However, at present these possibilities remain unexplored. What the in vitro studies did highlight was that the capacity of these cells to respond to ACTH was decidedly different in vitro compared to the in vivo situation—namely, in terms of basal versus ACTH stimulated cortisol production. This implicated additional intra- or extra-adrenal factors that in vivo act on the fetal adrenocortical cells limiting the capacity of low resting concentrations ACTH (5 to 10 pM) to stimulate cortisol production under basal conditions yet allow an enhanced cortisol production in response to a secondary stressor with stress levels of ACTH (50–100 pM). To address these possibilities, we explored two potential mechanisms that could potentially explain the noted adaptation of the fetal adrenal cortex to LTH: nitric oxide (NO) and leptin.

## 5. Role of Nitric Oxide in Mediating LTH Effects on Cortisol Biosynthesis

While the lower expression of the ACTH receptor, coupled with decreased expression of two rate limiting enzymes mediating cortisol biosynthesis (CYP11A1 and CYP17), provides a means for the capacity of the LTH fetus to maintain the normal ontogenic rise in fetal plasma cortisol, these adaptive changes seem contradictory to the capacity of the LTH adrenal cortex to produce greater cortisol in response to a secondary stressor. Although the enhanced ACTH_1–39_ released in the LTH fetus in response to a secondary stressor likely contributes to this boost in cortisol production, the decreased expression of the ACTH receptor coupled with lower expression of key enzymes suggests that other intracellular mechanism are involved in the divergent capacity for basal cortisol and stress-stimulated cortisol production.

**Figure 2:**
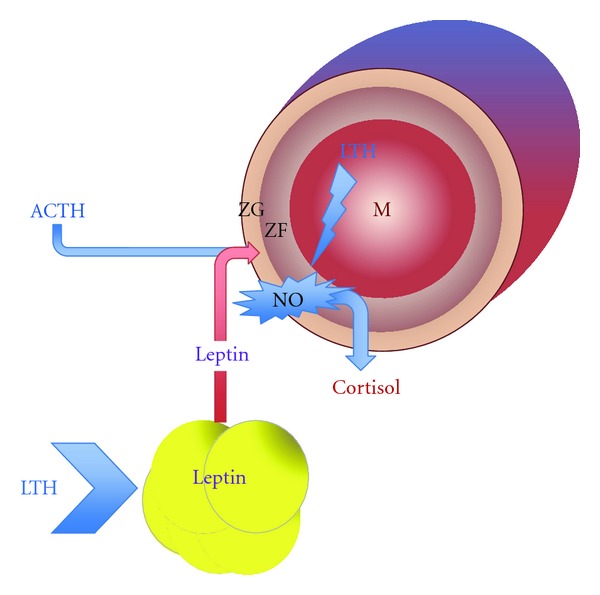
Effects of development under conditions of long-term hypoxia (LTH) on adrenocortical function in the late gestation ovine fetus. LTH increases perirenal adipose expression and release of leptin as well as adrenocortical leptin receptor (OB-Rb) expression. LTH also increases zona fasciculate-specific expression and activity of eNOS. Combined, leptin and nitric oxide (NO) serve to limit the ability of the elevated fetal plasma ACTH to stimulate cortisol production thus allowing for the maintenance of the normal ontogenic maturation of cortisol production preserving the prepartum exponential rise of this key steroid. In response to an acute secondary stress, these mechanisms allow release of cortisol synthesis thus resulting in an enhanced acute phase production of cortisol. (ZG-zona glomerulosa, ZF-zona fasciculata, M-medulla).

We initiated investigations into nitric oxide (NO) as one potential mediator of the effects of LTH on the adrenal gland. Nitric oxide has profound inhibitory effects on steroidogenesis in a wide range of endocrine tissues including the adrenal cortex and NO synthases (NOSs) are subject to regulation by hypoxia (see recent review by Ducsay and Myers [39]). As such NO represents a potential mechanism in the adrenocortical adaptation to LTH (Figure 2). Further, it has been reported that NO concentrations in the plasma of the LTH fetus are elevated compared to normoxic fetuses [42]. While the levels in plasma are likely not high enough to be of physiological importance for regulating intracellular adrenocortical events, they are indicative that NOS expression and/or activity may be elevated in target organs such as the adrenal gland.

Indeed, we were the first laboratory to demonstrate NOS expression in the fetal adrenal cortex and the effects of NO on fetal adrenal steroidogenesis. Endothelial NOS (eNOS) is the predominant NOS isoform in the ovine fetal adrenal cortex, and more importantly, we demonstrated that LTH increases eNOS mRNA and protein expression [43] and that the increased expression was primarily in CYP17 expressing cells within the zona fasciculata indicating a selective increase in the cortisol producing adrenocortical layer. In vitro studies demonstrated that enhancement of NO production with L-arginine, a NOS substrate, resulted in a significant reduction of ACTH-mediated cortisol production selectively in LTH-derived fetal adrenocortical cells [44]. The use of NO donors, DETA-NO, and Sodium Nitroprusside (SNP) similarly decreased ACTH-mediated cortisol production while inhibition of NOS activity with L-NAME significantly increased cortisol production in the LTH group but was without effect on normoxic fetus-derived fetal adrenocortical cells. In accord with the elevated expression on eNOS, nitric oxide synthase activity was significantly higher in LTH-derived fetal adrenocortical cells compared to those from normoxic control fetuses, and this difference was eliminated following ACTH treatment. These data indicate that LTH enhances adrenal cortical sensitivity to the inhibitory effects of NO on cortisol production. Nitric oxide may, therefore, play an important role in regulating ACTH-induced cortisol production in the LTH fetal adrenal [44].

How NO mediates its inhibitory actions on steroidogenic tissue, including the adrenal, is equivocal. In vascular tissue, the effects of NO are well established and largely depend on cGMP pathways (reviewed in [45]). However, the effects of NO on steroidogenesis in endocrine tissues appear to be independent of cGMP [46]. One potential mechanism by which NO inhibits steroidogenesis is via acting on the initial rate-limiting enzyme, CYP11A1. Nitric oxide competes with oxygen binding in the active site of CYP11A1 and CYP17 [46, 47]. Further, Peterson et al. [48] suggested that because these enzymes use several rounds of attack of the heme-oxygen complex on the steroid substrate, multistep enzymes (such as CYP11A1) would be more sensitive to NO inhibition than other steroidogenic enzymes. Overall, this competitive interaction makes NO a potentially effective inhibitor of steroidogenesis.

Thus, a model can be proposed where eNOS is upregulated in the LTH fetus, and under basal conditions (low ACTH), NO decreases the activity of CYP11A1 and perhaps other cytochrome P450 enzymes helping to limit the production of cortisol despite the elevated basal ACTH_1–39_ (Figure 2). During a secondary stress-induced elevation of ACTH, high levels of ACTH signaling inhibit NOS activity removing this inhibition resulting in the observed enhanced cortisol production observed in these fetuses. These findings implicate eNOS/NO as an intra-adrenal mechanism. Importantly the NO effects are also retained in vitro [43, 44]. Therefore, although NO plays a role in providing a brake on basal cortisol production, it does not appear to provide a mechanism by which the expression of key genes is decreased in the LTH adrenal gland. Taken together, these data implicate additional in vivo factors involved in the adaptation of the HPA axis to LTH.

## 6. Role of Leptin Mediating LTH Effects on Cortisol Biosynthesis

A role for leptin in suppressing the HPA axis in adults has been established, both at the hypothalamic level as well as directly at the level of the adrenal cortex. The active or long form of the leptin receptor (OB-Rb) is found in the adrenal cortex in rodents, humans [49], and ruminants [50]. Leptin suppresses cortisol output in response to ACTH stimulation in adult bovine adrenocortical cells, and this effect was mediated through an reduction in CYP17 expression [51]. Additional studies confirmed this observation and extended the inhibitory effect of leptin to expression of CYP11A [52]. In addition to downregulation of steroidogenic enzymes, leptin also appears to inhibit adrenal glucocorticoid secretion by an additional mechanism. Recent studies in the rat have clearly demonstrated that the leptin inhibition of corticosterone secretion occurs through a rapid reduction in expression of both StAR and PBR protein [53, 54].

Although the function(s) of leptin in the adult has been widely studied, the role of leptin in the fetus is not well defined. Clearly, leptin is found in the fetal circulation and is expressed in fetal adipocytes [55, 56] and placental trophoblast tissue [57]. Intracerebral infusion of leptin blunted the size of increase that occurred in amplitude and mean value of plasma ACTH and cortisol pulses near term [58]. These data are consistent with adult mouse data where leptin attenuates restraint-induced activation of the HPA [59]. More recent studies from the McMillen group also demonstrated an inhibitory effect of leptin on the fetal ovine HPA. Intravenous leptin infusions suppressed prepartum rise in both ACTH and cortisol [60]. Infusion after day 144 did not affect ACTH but there was still marked suppression of cortisol up to three days prior to delivery.

Based on this evidence, we initially addressed whether leptin, a hypoxia inducible gene, was elevated in LTH fetal sheep. We noted that plasma leptin as well as peri-renal adipose expression of leptin was significantly elevated in the LTH fetus as was expression of the OB-Rb in both the adrenal cortex and hypothalamus [61]. While past studies in the ovine fetus, and largely in adults as well, have focused on the actions of exogenous leptin in regulating the HPA axis, we have addressed the role of endogenous leptin in the HPA adaptation to LTH using a specific ovine leptin receptor antagonist. As we have recently communicated [62], a 4-day infusion of the antagonist to LTH fetuses starting at 139 days of gestation restored expression of both CYP11A1 and CYP17 to levels similar to those of the normoxic control fetuses. Surprisingly, the infusion of the leptin antagonist had no effect on expression of either enzyme in the normoxic controls and, similarly, did not alter fetal plasma ACTH or cortisol in these fetuses during the infusion period. In the LTH fetuses, although CYP11A1 and CYP17 expressions increased in response to the antagonist, fetal plasma cortisol levels were not altered and were not different from control fetuses. This indicates that, while leptin may play a role in mediating the adaptive responses to LTH in CYP expression in the adrenal gland, clearly other mechanisms exist, such as NO production, that limit the ability to produce cortisol. The lack of effect of the antagonist in the control fetuses would seemingly bring to question the role of leptin in regulating the HPA axis of the late gestation sheep fetus in a nonperturbed state. It should be noted that we have shown that the OB-Rb expression in the ovine fetal adrenal does decrease significantly near term compared to younger animals at ~120–130 days' gestation [63]. Thus, leptin may play a role in regulating the HPA axis in less mature fetuses and this regulation is lost near term as the HPA axis rapidly matures in preparation for birth (Figure 2).

## 7. HPA Response to LTH: Adaptive versus Maladaptive

 As discussed previously, we have defined changes in function of the fetal HPA axis in response to development under conditions of LTH as primarily adaptive, especially at the level of adrenal cortisol production. Indeed, these responses appear to support continued normal growth of the fetus in the face of the stress of sustained, moderate hypoxia. Considering the essential role of maturation of the fetal HPA axis and the late gestation exponential rise in fetal plasma cortisol in initiating parturition in this species, this adaptation also allows the fetus to remain in utero for the normal length of gestation. This seems especially critical considering the lowered oxygen delivery to the fetus, thus allowing the maximal opportunity for the fetal growth. As such, the observed response providing for the normal ontogenic maturation of adrenocortical function is critically adaptive in nature.

 However, the changes we have observed at the level of the hypothalamic PVN and anterior pituitary corticotrope may be viewed differently. Indeed, these changes might be argued as being maladaptive since they cumulatively result in enhanced processing of POMC to ACTH_1–39 _in the corticotrope and enhanced release of ACTH_1–39 _into the circulation. Under situations where there is no additional (acute secondary) stress, the adrenal cortex, perhaps in concert with adipose derived leptin, has developed mechanisms to maintain “normal” function. But, when presented with an acute secondary stress resulting in greater ACTH release, cortisol production is actually greater than that of age matched normoxic fetal sheep. Although cortisol production in response to stress is part of the physiological homeostatic mechanism(s) helping the organism to survive the stressor, it could be argued that the excessive production of cortisol in response to the secondary stress in the LTH fetus is maladaptive. This would be especially true in situations where the LTH-compromised fetus is subjected to repeated episodes of acute secondary stress or perhaps greatly amplified when a longer-term secondary stress occurs. In the final weeks of gestation, this capacity of the LTH fetus to produce a larger cortisol response to a secondary stress may serve as signal to terminate the pregnancy in this species indicative of a dangerously adverse intrauterine environment thus limiting the potential chance for fetal demise.

 Lastly, the changes in gene expression that we have noted in the perirenal adipose may also provide a means for the LTH newborn to survive in an adverse environment. Clearly, the increased production of leptin provides an essential brake to the adrenocortical response to LTH allowing the fetus to potentially remain in utero for a longer period. Perhaps more importantly however, the changes in gene expression in the adipose favor the brown fat phenotype that may aid the newborn LTH fetus in survival in the initial hours after birth. Similarly, the greater capacity for cortisol production in response to a secondary stressor, if retained post birth, would seem largely beneficial in a harsh, low oxygen environment.

## 8. Conclusions

Hypoxia, acute or sustained, is a major threat to the well-being of a fetus and is a relatively common insult during human pregnancy. During the final third of gestation the ovine fetal HPA axis undergoes a maturational process in which the fetus not only develops the capacity to mount a cortisol response to physiological stressors but also undergoes an exponential rise in basal cortisol. As with most mammals, this parturient rise in cortisol is essential for maturation of fetal organ systems allowing for survival in the extrauterine environment, after birth [64–66]. However, sustained or repetitious fetal stressors can activate the hypothalamic-pituitary-adrenocortical (HPA) axis resulting in elevated cortisol production and premature maturation of the adrenal cortex contributing to fetal growth restriction [67–69]. Nonetheless, the capacity to mount an adrenocortical stress response during late gestation contributes to fetal survival in the face of the stressor, as happens with adults. We have established a model of altitude-induced long-term hypoxia where the ovine fetus develops under continuous moderate hypoxia. Despite this constant physiological stressor, the fetal HPA axis undergoes a remarkable adaptation in which the normal ontogenic rise in fetal plasma cortisol and HPA axis maturation is maintained while preserving the capacity to mount a stress response. Indeed, the cortisol response to a secondary stressor is amplified, undoubtedly to aid in helping the fetus survive in this hypoxic environment. We have identified both adaptive responses at the level of the hypothalamus and anterior pituitary as well as the adrenal cortex that contribute to this adaptation to LTH and have identified two mechanisms, one intraadrenal (NO production) and extra-adrenal (adipose-derived leptin) that play significant roles in maintaining function of the fetal HPA axis at the level of the adrenal gland. It will be important to determine if these systems are a unique response to LTH or are invoked as a general adaptive response to other intrauterine stressors that aid in fetal survival.
